# High LEF1 expression predicts adverse prognosis in chronic lymphocytic leukemia and may be targeted by ethacrynic acid

**DOI:** 10.18632/oncotarget.7795

**Published:** 2016-02-29

**Authors:** Wei Wu, Huayuan Zhu, Yuan Fu, Wenyi Shen, Kourong Miao, Min Hong, Wei Xu, Lei Fan, Ken H. Young, Peng Liu, Jianyong Li

**Affiliations:** ^1^ Department of Hematology, the First Affiliated Hospital of Nanjing Medical University, Jiangsu Province Hospital, Collaborative Innovation Center For Cancer Personalized Medicine, Nanjing, China; ^2^ Department of Hematopathology, The University of Texas MD Anderson Cancer Center, Houston, TX, USA; ^3^ Department of Hematology, Zhongshan Hospital, Fudan University, Shanghai, China

**Keywords:** chronic lymphocytic leukemia, LEF1, CYLD, necroptosis, ethacrynic acid

## Abstract

Aberrant activation of lymphoid enhancer-binding factor-1 (LEF1) has been identified in several cancers, including chronic lymphocytic leukemia (CLL). As a key transcription factor of the Wnt/β-catenin pathway, LEF1 helps to regulate important genes involved in tumor cell death mechanisms. In this study, we determined LEF1 gene expression levels in CLL (*n* = 197) and monoclonal B-cell lymphocytosis (MBL) (*n* = 6) patients through real-time RT-PCR. LEF1 was significantly up-regulated in both MBL and CLL patients compared with normal B cells. Treatment-free survival (TFS) time and overall survival (OS) time were much longer in CLL patients with low LEF1 expression than in those with high LEF1 levels. Furthermore, Wnt inhibitor ethacrynic acid (EA) induced both apoptosis and necroptosis in primary CLL cells. EA also enhanced the cytotoxicity of both fludarabine and cyclophosphamide against CLL cells in vitro. Finally, we demonstrated that EA functions by inhibiting the recruitment of LEF1 to DNA promoters and restoring cylindromatosis (CYLD) expression in CLL cells. Our results showed, for the first time, that high LEF1 expression is associated with poor survival for CLL patients. Combined with other chemotherapeutic drugs, EA may be a promising therapeutic agent for CLL.

## INTRODUCTION

Chronic lymphocytic leukemia (CLL) is a B-cell hematological malignancy characterized by the clonal expansion and accumulation of morphologically mature B-lymphocytes in peripheral blood, bone marrow, and secondary lymphoid tissues. The progressive accumulation of leukemic cells is mostly ascribed to extended cellular survival rather than excessive cellular proliferation [1]. CLL is highly heterogeneous in clinic and remains incurable at present. Thus, improving the risk-stratification system for CLL and investigating more effective therapeutic agents are great challenges for the future management of CLL. Investigation of aberrantly activated genes with prognostic relevance in CLL may help to identify those patients with a poorer prognosis and to develop novel therapeutic targets for CLL.

Lymphoid enhancer binding factor-1 (LEF1) is a member of the LEF/T-cell factor (TCF) family. As a central mediator of the canonical wingless-type (Wnt) signaling pathway, LEF1 regulates a variety of genes related to cell cycle regulation and cellular proliferation [2]. Recent data demonstrated a vital role of LEF1 in early hematopoiesis and leukemic transformation in murine models [3]. LEF1 also plays a critical role in normal human hematopoiesis, especially in the development of B- and T-lymphocytes [4,5]. Aberrant expression of LEF1 has been found in several hematological malignancies [6-12] in human. High LEF1 expression is associated with favorable prognosis in acute promyelocytic leukemia (APL) and cytogenetically normal acute myeloid leukemia (AML) [8,9], while it is a negative prognostic marker in adult B precursor acute lymphoblastic leukemia (B-ALL) [10]. However, the prognostic significance of LEF1 expression has not been thoroughly clarified in CLL.

Cylindromatosis (CYLD) is a deubiquitinazing enzyme that regulates a variety of physiological processes, including cellular mitosis, B-cell homeostasis, immune response and T cell development [13]. Previous study has proved CYLD as a tumor suppressor gene. Loss of CYLD inhibits cellular apoptosis by activating NF-κB pathway [14]. Moreover, CYLD is a key regulator in necroptosis, which is a recently coined term used to describe one particular form of programmed necrosis induced by stimulating death receptors [15]. CYLD knockdown inhibits cellular necroptosis induced by TNF-α in Jurkat cells [16]. Our group has previously proved that CLL cells have defects in necroptotic signaling. We have also identified LEF1 as a transcriptional repressor of CYLD in CLL cells and inhibition of LEF1 expression sensitizes CLL cells to necroptosis [17]. Thus, targeting the LEF1-CYLD axis to restore the necroptotic pathway may represent a novel approach for CLL treatment.

Previous studies have demonstrated that ethacrynic acid (EA), a loop diuretic drug once commonly used in clinical practice, was cytotoxic to primary CLL cells, myeloid leukemia cell lines, and other types of cancer cell lines [18-22]. As an antagonist of Wnt signaling pathway, EA selectively induced cellular death in primary CLL cells at a low concentration by inhibiting Wnt signaling [23].

In the present study, we analyzed LEF1 expression in a large cohort of CLL patients and identified LEF1 as an adverse prognostic factor in CLL patients. In addition, we found that EA induces both apoptosis and necroptosis in CLL cells *in vitro*. We demonstrated that EA suppresses cell survival by inhibiting the recruitment of LEF1 to DNA promoters and restoring CYLD expression in CLL cells.

## RESULTS

### LEF1 expression is aberrantly overexpressed in MBL and CLL patients

**Figure 1:**
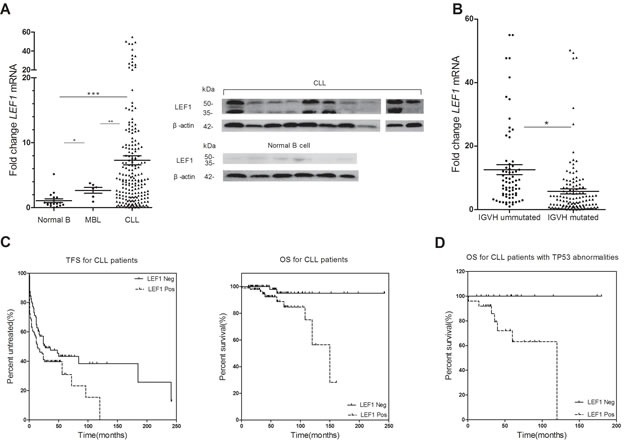
The prognostic significance of LEF1 for CLL patients (**A.**, right) LEF1 mRNA expressions of 197 CLL patients, 6 MBL patients, and 18 normal B-cell samples have been determined using real-time quantitative RT-PCR. The horizontal lines represent the mean value of the group. *P = 0.0154, **P < 0.0001, ***P < 0.0001. (**A.**, left) Western blot analysis of LEF1 in 10 CLL patients and 6 normal B-cell samples. β-actin was used as a loading control. **B.** Dot-plot of LEF1 mRNA expression in CLL patients with mutated or ummutated IGHV. The mean expression of LEF1 is much higher in ummutated cases compared with mutated cases. *P = 0.0002. (**C.**, right) Time free survival (TFS) was measured from the time of diagnosis. High level of LEF1 mRNA expression indicates a shorter TFS. The median TFS for LEF1-positive patients was 14 months, while the median TFS for LEF1-negative patients was 27 months. *P =* 0.0356. (**C.**, left) Overall survival (OS) was determined from the time of diagnosis. The median OS for LEF1-positive patients was 150 months; the median OS for LEF1-negative patients was not reached. *P =* 0.0030. **D.** LEF1 was an adverse prognostic factor linked to inferior OS in patients carrying 17p deletion or TP53 mutation. The median OS for LEF1-positive patients was 120 months; the median OS for LEF1-negative patients was not reached. *P =* 0.0066.

We assessed LEF1 mRNA expression in 197 samples from CLL patients, 6 samples from MBL patients, and 18 samples from healthy donors. As shown in Figure 1A (left), CLL cells exhibited a dramatically stronger expression of LEF1 mRNA than did normal B-cells (*P* < 0.0001). LEF1 mRNA expression in MBL cells was also stronger than in normal B cells (*P* = 0.0154). We then used Western blot to assess LEF1 protein expressions in samples from 10 CLL patients and 6 healthy donors. The data confirmed that LEF1 protein levels in CLL cells were significantly up-regulated over that in normal B-cells (Figure 1A, right). These results indicated a role for LEF1 in CLL leukemogenesis, so we wished to further investigate the potential prognostic effect of LEF1 expression in CLL patients.

### The prognostic significance of LEF1 in CLL patients

We established correlations between LEF1 expression level and clinical characteristics in CLL patients. The characteristics of CLL patients are listed in Table1. Our analysis revealed that high LEF1 expression was strongly associated with unmutated IGHV status (*P =* 0.0002; Figure 1B). Patients with unmutated IGHV have a mean LEF1 expression of 12.56, whereas those with mutated IGHV have a significantly lower mean LEF1 expression of 5.75. No correlation was found between LEF1 expression and other clinical, molecular, or cytogenetic features. Of note, we also did not observe any significant association between LEF1 expression and ZAP70 or CD38 levels.

**Table 1:** CLL patient cohort features

	All patients (n=197)	LEF1 positive (n=99)	LEF1 negative (n=98)	P^*^
Median age, y(range)	63(19-81)	64(19-92)	62(35-81)	
Gender				0.4437
Male, n	136	71	65	
Female, n	61	28	33	
*IGHV* somatic mutation status(n=176)^∞^				< 0.0001
Mutated, n	117	48	69	
Unmutated, n	59	44	15	
ZAP70 protein level(n=176)^#^				1.0000
Positive, n	44	23	21	
Negative, n	132	67	64	
CD38 protein level(n=176)^§^				0.3098
Positive, n	48	20	28	
Negative, n	128	66	62	
Cytogenetic features(n=178)				0.4383
None, n	98	49	49	
Isolated del(13q), n	26	12	14	
Unfavorable abnormalities, n	54	32	22	
*TP53* status (n=150)^**^				0.3588
*TP53* abnormal, n	40	24	16	
*TP53* normal, n	110	56	54	
Rai stage				1.0000
0-2, n	124	61	60	
3-4, n	73	37	36	
Binet stage				0.7411
A, n	85	42	43	
B, n	41	19	22	
C, n	67	36	31	
Patients requiring no treatment, n	80	36	44	0.2475
Patients requiring treatment, n	117	63	54	
Patients still alive, n	184	87	97	0.0101
Patients died during study, n	13	11	2	

* Two-sided Fisher's exact test was performed in this section, and a *P* value≤0.05 was considered statistically significant.

∞ Mutated *IGHV* is according to a 98% cutoff value.

# *ZAP70* positive samples have >20% *ZAP70* expression cells.

§ *CD38* positive samples have >30% *CD38* expression cells.

** *TP53* abnormalities include patients with 17p deletion or *TP53* mutation.

For survival analysis, patients were divided into high- and low-LEF1 subgroups by LEF1 expression level median split. Both treatment-free survival (TFS) time and overall survival (OS) time were much shorter in the high-LEF1 subgroup than in the low-LEF1 subgroup (Median TFS: LEF1 high, 27 months, LEF1 low, 14 months, *P* = 0.0356, Figure 1C, left; Median OS: LEF1 high, 150 months, LEF1 low, not reached, *P* = 0.003, Figure 1C, right). Additionally, we found that for patients with TP53 mutation or 17p deletion, high LEF1 expression is predictive for inferior OS (Median OS: LEF1 high, 120 months, LEF1 low, not reached, *P* = 0.0066, Figure 1D).

### EA suppressed the expressions of Wnt target genes in CLL cells

**Figure 2:**
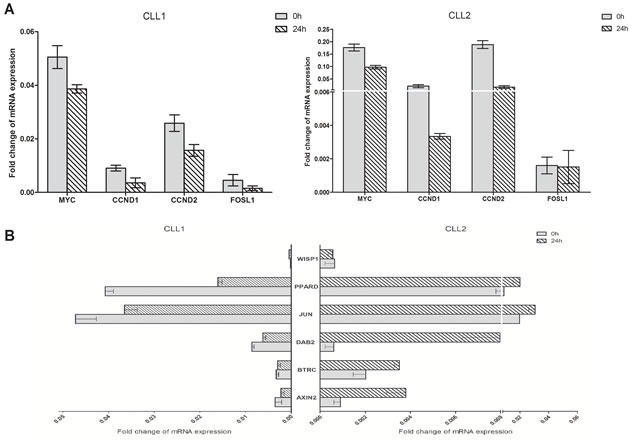
EA suppressed the expressions of Wnt target genes in CLL cells Primary cells from two CLL patients were collected and treated with 10μM EA for 24 hours. Realtime-PCR was employed to detect the mRNA expressions of Wnt target genes. The relative fold change was normalized against GAPDH.

To investigate the antagonistic effect of EA on Wnt signaling in CLL cells, the expression levels of Wnt target genes were detected. Primary CLL cells from 2 patients were collected and incubated in 10μM EA for 24h. The mRNA expressions of 10 commonly established Wnt target genes were examined using real-time PCR. As shown in Figure 2A, the expression of CCND1, CCND2, MYC and FOSL1 were down-regulated in both samples after EA incubation. Among these 4 genes, CCND1 and MYC are well identified target genes of LEF1. No coherent trend in expression alterations has been found in both samples for the other 6 genes (Figure 2B). Thus, our results indicate that EA inhibits Wnt signaling in CLL cells, and the function of LEF1 was impaired by EA.

### EA induced both apoptosis and necroptosis in CLL cells and enhanced the cytotoxicity of chemotherapeutic agents *in vitro*

**Figure 3:**
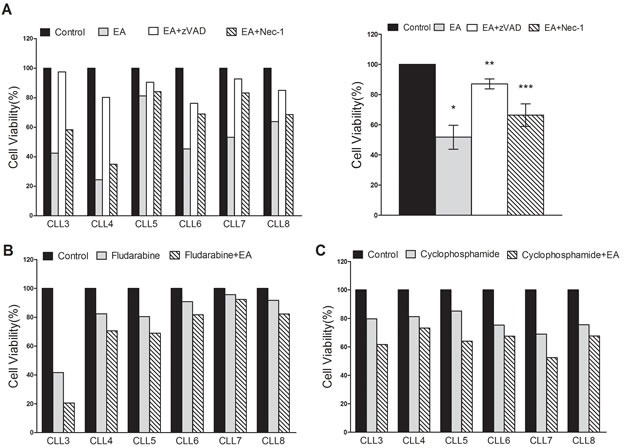
EA induced apoptosis and necroptosis in CLL cells and accelerated efficacies of fludarabine and cyclophosphamide *in vitro* **A.** Primary CLL cells from six patients were treated with pure medium, EA, EA+zVAD and EA+Nec-1. Cell viabilities at 48 hours after treatment from six CLL patients were obtained through MTT assay (left panel). The average change of cell viabilities was shown in right panel. Horizontal line represents the mean value of the group. The error bar represents the SEM. *P = 0.0017, **P = 0.0056, ***P = 0.0206. **B.** Primary CLL cells were treated with fludarabine with or without 10μM EA. **C.** CLL cells from the same six patients were treated with cyclophosphamide with or without 10μM EA. Cell viability was measured by MTT assay.

We proceeded to demonstrate the effect of EA on primary CLL cells. Each sample was divided into four treatment groups and treated with pure medium, EA (10 μM) alone, EA plus benzyloxycarbonyl-Val-Ala-Asp-fluoromethylketone (zVAD, 20 μM), or EA plus Necrostatin-1 (Nec-1, 30 μM), respectively. Cell viabilities were examined by MTT at 48h. As shown in Figure 3A, EA treatment led to remarkable decrease in CLL cell viabilities. Interestingly, the cytotoxic effect of EA was inhibited both by zVAD and Nec-1. As zVAD is a classic pan-caspase inhibitor, the result suggested that EA has induced apoptosis in CLL. Given that Nec-1 is a specific inhibitor of necroptosis, our finding also demonstrated that EA led to necroptosis in CLL as well. After the cell viability data from all six CLL patients were analyzed together, we found that Nec-1 saved a smaller proportion of CLL cells from the effects of EA than did zVAD, indicating necroptosis contributed to a relatively small proportion of cell deaths induced by EA. To better evaluate the effect of EA on necroptotic pathway in CLL cells, we treated cells with TNFα, a cytokine that promotes inflammatory response (30 ng/ml), plus zVAD (20 μM) with or without EA (10 μM). As expected, EA treatment led to a remarkable increase in CLL necroptosis in response to treatment with TNFα plus zVAD (data not shown). We then sought to determine whether EA could strengthen the effect of agents presently used in chemotherapies for CLL. Primary CLL cells were treated with fludarabine or cyclophosphamide for 24h either with or without EA. As shown in Figure 3B and 3C, EA promoted the effect of fludarabine most significantly in patients 3, 4, and 5 (labeled CLL 3, CLL4, and CLL5, respectively, on the graph) whereas no obvious improvement was observed in patient7. On the other hand, EA dramatically enhanced cyclophosphamide's cytotoxicity for all six patients. Collectively, our results suggest that EA may restore CLL cells' vulnerability to chemotherapeutic agents through apoptotic and necroptotic pathways.

### EA interfered with LEF1 binding to DNA and restored CYLD expression

**Figure 4:**
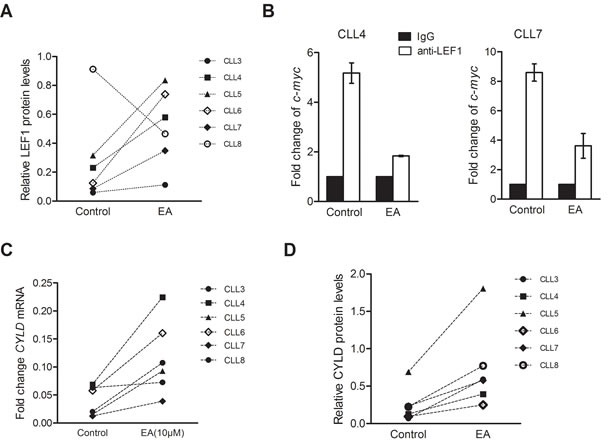
EA inhibited LEF1 recruitment to DNA and restored CYLD expression **A.** LEF1 protein levels from the same six patients as mentioned above were detected with western blot after 10μM EA incubation for 24 hours. CLL cells from five patients showed enhanced expressions of LEF1, while only one sample (CLL6) showed a repressed LEF1 expression after EA treatment. **B.** ChIP assay was performed to determine the binding of *LEF1* to DNA promoters. The ChIP DNA was amplified with primers flanking the human *c-myc* promoter that contains LEF1 binding sites. IgG was used as a control in this data. **C.** CYLD mRNA levels after EA incubation were determined by Q-PCR in the six CLL patients. **D.** CYLD protein levels after EA incubation were examined by western blot in the same six CLL patients.

We proceeded to explore the effect of EA on LEF1. Primary CLL cells were incubated in pure medium and 10μM EA for 24h. Cells were collected for quantitative RT-PCR or Western blot. Unexpectedly, EA enhanced LEF1 expressions in five of six primary samples. Only one sample showed decreased LEF1 expression after EA treatment. The LEF1 protein expression changes are shown in Figure 4A. As EA has decreased CLL cell viabilities in all six samples, it is possible that EA does not work by suppressing LEF1 expression. We then investigated if EA had led to LEF1 dysfunction. Binding to DNA is a key step in LEF1's regulation of target genes. To find out whether EA would inhibit the recruitment of LEF1 to DNA promoters, we performed a ChIP assay on primary CLL cells. The DNA/protein complex was pulled down using anti-LEF1 antibody. The purified DNA was amplified with primers flanking the human *c-myc* promoter that contains LEF1 binding sites. As shown in Figure 4B, LEF1 recruitment was inhibited in CLL cells treated with EA. Our team has previously demonstrated that LEF1 is a transcriptional repressor of CYLD in CLL. Thus, we hypothesized that EA would enhance CYLD expressions by restricting LEF1 function. We detected CYLD expression alterations in CLL cells from the six patients mentioned above. As shown in Figure 4C and 4D, EA incubation resulted in dramatic elevations in CYLD levels in all six samples, as determined by quantitative PCR and western blot.

### Knocking down CYLD partially inhibits EA cytoxicity to CLL cells

**Figure 5:**
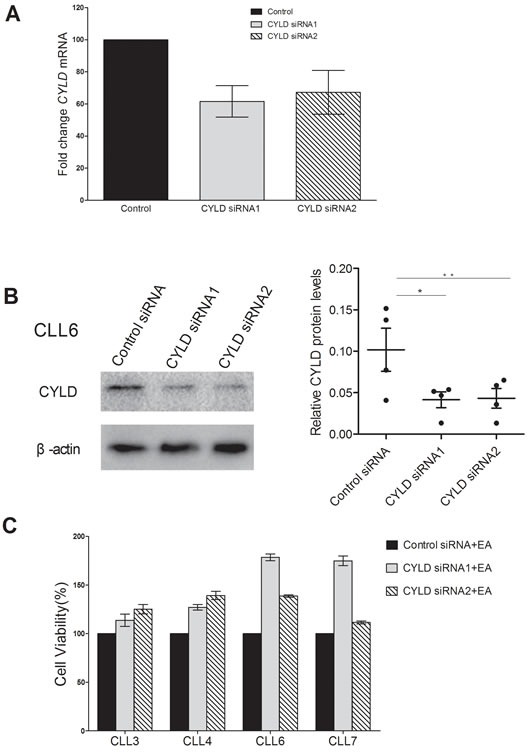
Knocking down of CYLD partially inhibited EA cytotoxicity on CLL cells **A.** CYLD mRNA expression was determined by real-time RT-PCR 24 hours after transfection. Relative fold changes from four CLL patients were normalized to control siRNA. **B.** Western blot analysis was performed to detect CYLD protein levels 48 hours after siRNA treatment. Horizontal line indicates the mean value of the group. Error bar represents SEM. *P = 0.0500, **P = 0.0288. **C.** CLL cell were transfected with siRNA targeting CYLD for 48 hours, followed by 10μM EA incubation for another 24 hours. Cell viability was accessed by MTT assay.

Finally, we sought to examine the effect of CYLD inhibition on the cytotoxicity of EA to CLL cells. Two separate siRNAs targeting CYLD were used in the transfection. CLL cells were transfected with siRNAs targeting CYLD for 48h followed by 10μM EA incubation. CYLD expressions were dramatically decreased at mRNA and protein levels, as determined by quantitative RT-PCR and Western blot (Figure 5A and 5B). As shown in Figure 5C, CYLD knockdown led to a remarkable protection against EA's cytoxicity on CLL cells compared with control siRNA. Collectively, the results suggest that CYLD has participated in EA-induced cell death in CLL.

## DISCUSSION

In the current study, we have identified the overexpression of LEF1 in CD5+ B cells from MBL and CLL patients compared to those from healthy donors. The result is consistent with previous reports from our group and other investigators [7, 17, 25]. LEF1 is a nuclear mediator of the canonical Wnt pathway and plays a regulatory role in lymphoid proliferation and differentiation. In mice, LEF1 expression is restricted to pre-B and pro-B cells [5]. A similar pattern of LEF1 expression has been identified in human. It has been found that LEF1 is expressed by human B cell precursors, while its expression is lost in mature B cells [7]. Dysregulation of LEF1 leads to B lymphoblastic in murine models [3]. Inhibition of Wnt /β-catenin/LEF1 pathway has been found to induce apoptosis in CLL cells [26]. Likewise, LEF1 knockdown leads to TNF-α induced necroptosis in CLL cells [17]. In this respect, the reacquiring of LEF1 expression by neoplastic B cells may contribute to the prolonged survival of CLL cells, indicating a pathogenetic role of LEF1 in CLL.

In further investigations, we have identified a marked association between high LEF1 expression and IGHV unmutated status. We also show, for the first time, that high LEF1 expression is linked to inferior OS in CLL patients, especially those with TP53 abnormalities. IGHV mutational status is one of the strongest independent risk factors in CLL and gives prognostic information irrespective of clinical stages. The relatively high expression level of LEF1 in IGHV unmutated cases suggests a role of LEF1 in aggressive clinical behavior of CLL. ZAP70 protein expression has been reported to correlate with somatic mutation status of IGHV. However, no statistically significant relevance has been found between LEF1 expression and ZAP70 positivity in our study. One possible explanation for our result is ZAP70 protein expression is not strictly restricted to unmutated IGHV status [27]. Another interpretation would be the variance of ZAP70 protein expression level assessed by flow cytometric analysis [28]. Interestingly, Erdferder *et al.* found that LEF1 expression level is significantly higher in ZAP70-positive patients in comparison with ZAP70-negative patients [25]. However, they did not investigate the association between LEF1 expression and IGHV mutational status or the overall survival of CLL patients in the study. The discordant results may be due to the larger cohort of CLL patients from our study and genetic variance between different racial cohorts.

The presence of TP53 abnormality identifies a subgroup of CLL patients with aggressive clinical behavior, poor response to chemotherapies, and inferior outcome. We have observed significant prognostic difference between high LEF1 and low LEF1 expressers within this subgroup of patients. Our result indicates that the prosurvival effect of LEF1 is independent of TP53 abnormalities. Inhibition of LEF1 expression may circumvent the apoptosis evasion led by TP53 abnormalities in CLL cells. In this regard, LEF1 is a valuable therapeutic target for CLL treatment, especially for CLL patients harboring TP53 abnormalities.

Ethacrynic acid (EA) is a loop diuretic with an excellent safety profile. In the present study, we have found that EA induced both apoptosis and necroptosis in primary CLL cells. Apoptosis was the dominant type of cell death, as the protection provided by caspase inhibitor was greater than that provided by Nec-1. Furthermore, we have proved that when apoptotic mechanism was blocked, EA enhanced the TNFα-induced necroptotic pathway as a backup mechanism, ensuring the elimination of CLL cells. Previous studies have found that diphtheria toxin GM-CSF (DT-GMCSF) and shikonin could trigger both apoptosis and necroptosis in AML cell lines [29] and HL60 cells [30], and blocking one mechanism would force tumor cells to die through the other pathway. In this regard, the simultaneous activation of both mechanisms in primary CLL cells by EA would enhance the killing effect on tumor cells and prevent tumor cells from evolving drug resistance.

Lu and colleagues have identified EA as a specific antagonist of Wnt signaling [23]. We examined the expressions of Wnt target genes to further testify the inhibitory effect of EA on Wnt signaling in CLL cells. Noteworthy, EA inhibited the expression of CCND1 and MYC, both of which are target genes of LEF1. The inhibition of both LEF1 target genes suggested that the function of LEF1 was impaired by EA. In the following part, we examined the effect of EA on LEF1 expression in primary CLL cells from 6 patients. As shown by the real-time PCR and Western blot results, EA treatment led to up-regulations of LEF1 in 5 out of 6 samples, while only one CLL sample (CLL8) exhibited down-regulation of LEF1 expression after EA treatment. Compared to the other 5 patients, the distinctive patient had 3 particular features 1) extremely high white blood cell count (426.63×10^9^/L) in peripheral blood at diagnosis, 2) unmutated IGHV statues, and 3) the highest LEF1 expression level among 6 patients. It is possible that the effect of EA on LEF1 expression partially depends on the initial LEF1 expression level of CLL cells. Given that CYLD is a downstream target gene of LEF1, we then investigated the impact of EA treatment on CYLD expression. Interestingly, we found that EA enhanced CYLD expression in all 6 samples. Subsequent ChIP assay investigation has demonstrated that EA inhibited the recruitment of EA to DNA promoters in CLL cells. Thus we speculated that the enhancement of CYLD expression was due to the failed regulatory function for LEF1 in CLL cells. β-catenin/LEF1 signaling is activated in CLL cells, and inhibition of β-catenin/LEF1 interaction has been proved to induce apoptosis in CLL cells [26]. It has been reported that EA could directly bind to LEF1 protein and destabilize β-catenin/LEF1 complex in SW480 cells. Thus, the failed regulatory function for LEF1 in the present study may be due to the same effect of EA in CLL cells.

CYLD was initially identified as a gene mutated in familial cyclindromatosis. Owing to its role as a deubiquinase, CYLD has been associated with multiple kinds of hematological malignancies, including multiple myeloma, T-cell acute lymphoblastic leukemia (T-ALL), and CLL [31-34]. CYLD functions as a tumor suppressor through negative regulation of NF-κB pathway. NF-κB pathway is an anti-apoptotic pathway which is constitutively activated in CLL. Inhibition of NF-κB pathway induces apoptosis in CLL cells. Recent investigations have revealed the key regulatory role of CYLD in TNFα-induced necroptotic signaling in Jurkat cells [16]. We have demonstrated previously that CLL cells are defective in necroptosis and could recapture necroptotic response when CYLD expression has been restored [17]. Here we showed that knockdown of CYLD significantly protected CLL cells from EA-induced cell death, suggesting that CYLD may participate in the cytotoxic effect of EA on CLL cells. Moreover, based on the regulatory role of CYLD in NF-κB pathway and necroptotic signaling, it is tempting to speculate that the simultaneous activation of apoptosis and necroptosis by EA may be related to the enhanced expression of CYLD in CLL cells.

EA has been shown to be cytotoxic to multiple kinds of tumor cells, including CLL cells, and accelerate the cytotoxicity of chemotherapeutic agents. It has been found that EA increased the efficacy of lenalidomide to myeloma cells *in vitro* [35]. Jing and colleagues have proved that EA enhanced apoptosis in lymphoma cell lines treated by Arsenic Trioxide [20]. Here we showed that EA increased the cytotoxicity of fludarabine and cyclophosphamide against CLL cells. Moreover, EA has been found to partially reverse chlorambucil resistance in a B-CLL patient *in vivo* [36]. Drug resistance is a stubborn problem in CLL treatment. Drug resistance is relevant to multiple factors, among which the loss of p53 gene is a major contributor. Necroptotic signaling is distinct from apoptosis. Previous investigations have demonstrated that necroptosis could circumvent drug resistance resulted from anti-apoptotic protein overexpression and p53 loss in tumor cell lines [37, 38]. Here we have shown EA stimulated apoptosis and necroptosis in CLL cells, and the necroptotic pathway was enhanced when apoptosis was blocked. The simultaneous activation of both mechanisms by EA could ensure the efficacy of cytotoxicity to CLL cells and prevent tumor cells from evolving drug resistance. O'Dwyer and his colleagues have conducted a phase I trial of EA among patients with advanced solid tumors [39, 40]. According to the trial, the major adverse effect of EA was diuresis, which was manageable with proper monitoring, suggesting an outstanding safety profile of EA. Therefore, EA is a potential therapeutic agent for CLL patients when used alone or combined with other chemotherapeutic agents.

In conclusion, our investigation has provided additional strong evidence for the aberrant activation of LEF1 in MBL and CLL cells and has for the first time identified LEF1 as an adverse prognostic marker in CLL patients. These results indicate LEF1 may serve as an attractive therapeutic target for future CLL therapies. We have also demonstrated that EA induced both apoptosis and necroptosis in CLL cells by inhibiting LEF1 function and restoring CYLD expression. In addition, we have found that EA enhances the efficacy of chemotherapeutic agents to CLL cells *in vitro*. Our data suggests that EA is potentially an effective agent for CLL patients when used alone or combined with other chemotherapeutic agents.

## MATERIALS AND METHODS

### Patient and health donor samples

We obtained peripheral blood samples from 197 patients with CLL, 6 patients with MBL, and 18 healthy donors after obtaining informed consent between 2008 and 2012. The study was conducted according to the Declaration of Helsinki, after the approval from the Institutional Review Board of the First Affiliated Hospital of Nanjing Medical University. All patients were diagnosed according to the World Health Organization criteria. CLL patients were either previously untreated or had received no treatment for at least 6 months before the investigation.

### Cell isolation and culture

Mononuclear cells from CLL patients were isolated from peripheral blood using Ficoll density gradient centrifugation and purified by immunomagnetic separation with CD19 microbeads (Millipore) to obtain the CD19+ B-lymphocytes. Flow cytometric analysis was used to confirm that at least 95% of the CLL or normal B cells were positive for CD19 and negative for CD3. CLL cells for culture were resuspended in RPIM 1640 with 10% fetal bovine serum and maintained at 37°C in an atmosphere containing 5% CO_2_.

### Nucleic acid preparation

Total RNA was extracted from cells using Trizol (Invitrogen) according to the manufacturer's instructions. RNA quality was assessed using a spectrophotometer (BioRad). First-strand cDNA was synthesized by M-*MLV* Reverse Transcriptase (Invitrogen) using 1 microgram of total RNA. DNA was extracted from peripheral blood samples using a DNA isolation kit (Invitrogen) according to the manufacturer's instructions.

### Fluorescence *in situ* hybridization

Fluorescence *in situ* hybridization(FISH) analysis was performed on CLL cells to detect the cytogenetic abnormalities of certain chromosomal regions, including 6q, 11q, 13q, 14q, 17p, and chromosome 12. The fluorescent-labeled probes used in the present investigation are listed as follows: LSIMYB (6q23), LSI ATM (11q22), LSI D13S319 (13q14), LSI IGHC/IGHV (14q32), LSI p53 (17p13), and CEP12 (centromere 12; all the probes were purchased from Vysis, Downers Grove, IL, USA). 100 CLL cells were examined for each sample, and the percentage of cells with certain chromosomal abnormality was recorded. The identification of del(6q23) or del(11q22) in more than 7.5% of cells was considered positive. More than 10% of cells were required for determination of del(13q14) positivity, and more than 8.9% of cells for determining del(11q22) positivity. The determination of del(17p13) and trisomy 12 positivity needed more than 5% and 3% of cells, respectively.

### Evaluation of gene mutation status

Polymerase chain reaction(PCR) was performed in a 20-μL system containing 200 ng DNA, 0.25 μL DNA polymerase, 20 μM forward and reverse primer. The primers used for Notch1 amplification are listed in Table S1. PCR products were purified and sequenced in both directions. SeqMan software was used to analyze the sequencing data. The mutation status of *TP53* and B-cell receptor heavy chain gene (IGHV*)* were accessed as previously described [24].

### Quantitative reverse transcription-polymerase chain reaction

The forward and reverse primers were designed by Primer3:

LEF1 forward-,5′-AGAACACCCCGATGACGGA-3′, reverse-5′-GAGGGTCCCTTGTTGTAGAGG-3′;

CYLD forward-5′-AATGCAGCGTTACAGACAAACA-3′, reverse-5′-ACTTCCCTTCGGTACTTTAAGGA-3′;

β-actin forward-5′-TGACGTGGACATCCGCAAAG-3′; reverse-5′-CTGGAAGGTGGACAGCGAGG-3.

Quantitative reverse transcription-polymerase chain reaction (RT-PCR) was performed in a total volume of 20 μL, containing 1 μL of cDNA, 0.5 μM of each primer, 0.5 μM of SYBR Green dye, and 2 × master mix. Amplification was performed on the Mx3005P QPCR system as follows: denaturation at 95°C for 5 minutes, 40 cycles of 15 seconds at 95°C, 45 seconds at 58°C to 60°C, and 45 seconds at 72°C, followed by a melting curve cycle. Quantitative data was analyzed using Mx3005P software. A duplicate was set for each sample, and a mean of the cycle numbers of the two replicates was used in the analysis. The relative fold change was normalized against *β-actin.* The relative expression level was calculated using the 2^−△△CT^ method.

### Transfection and siRNA

Transfection was carried out using Liposome 2000 (Invitrogen) following the recommended protocols. siRNAs targeting *LEF1* were designed to inhibit the expression of full-length *LEF1* and the △N isoform. Cells were collected from 12 to 72 hours after transfection for further processions.

### Assessment of cell viability by 3-[4,5-Dimethylthiazol-2-yl]-2,5-diphenyl Tetrazolium Bromide (MTT)

Cell viability was assessed by MTT assay. CLL cells were resuspended and plated in 96-well plates at 2.5×10^5^ per well, followed by different treatments. Cells were then incubated at 37°C in the dark for 4 hours after 5mg/ml MTT had been added. 150μL Dimethyl sulfoxide (DMSO) was added to each well after the incubation. Optical densities at 490nm were read by Multiskan Ascent (BioRad). Four duplicates were set for each sample.

### Western blot

Fresh cells were lysed on ice for 30 minutes using lysis buffer containing 50 mM Tris (pH 7.4), 150 mM NaCl, 1% NP-40, 0.5% sodium deoxycholate, 0.1% sodium dodecyl sulfate, and 1 mM Ethylenediaminetetraacetic acid disodium salt (EDTA). The protein concentration was examined using the bicinchonininc acid (BCA) protein assay kit (Thermol). Proteins were loaded on 10% sodium dodecyl sulfate-polyacrylamide gel electrophoresis (SDS-PAGE) gels and transferred to polyvinylidene difluoride membranes (Millipore). The membrane was blocked in the blocking buffer included 0.1 M Tris-HCL, 0.9% NaCl, 0.8% KCl, 0.5% Tween-20 (TBST) and 5% nonfat silk milk and incubated with antibodies against human LEF1 (Cell Signaling Technology), β-actin (Cell Signaling Technology) at a 1:1000 dilution and CYLD (Cell Signaling Technology) at a 1:250 dilution, followed by incubation with goat anti-rabbit IgG antibody conjugated to horseradish peroxidase (Cell Signaling Technology) at a dilution of 1:5000. Enhanced chemiluminescence was used to detect proteins.

### Chromatin immunoprecipitation (ChIP) assay

ChIP assays were carried out according to the protocol for the ChIP Assay Kit (Upstate Biotechnology). Cells were cross-linked by 1% formaldehyde for 10 minutes at 37°C and the cross-linking was blocked by the addition of 125 mM glycine. Chromatin extracts containing DNA fragments were immunoprecipitated by the anti*-*LEF1 antibody (Santa Cruz). Protein A was used to collect the antibody/histone complex. ChIP DNA was recovered and then amplified with primers flanking the human *c-myc* promoter that contains *LEF1* binding sites. All ChIP assays were repeated at least 3 times.

### Statistical methods

To analyze the correlation between LEF1 expression levels and clinical characteristics of CLL patients, the nonparametric Mann-Whitney U test and two-sided Fisher's exact test were performed. Survival curves were calculated by the Kaplan-Meier-method using the log-rank test. For the data-staining comparisons between treated and control groups, a paired t-test or two-way ANOVA analysis was performed. A *P* value ≤ 0.05 was considered statistically significant. SPSS software version 11.0 was used for all analysis.

## SUPPLEMENTARY MATERIAL TABLE AND FIGURE


