# Supplementary material for: High LEF1 expression predicts adverse prognosis in chronic lymphocytic leukemia and may be targeted by ethacrynic acid
Source: Oncotarget. 2016 Feb 29;7(16):21631–43. doi: 10.18632/oncotarget.7795 (PMC5008311; doi:10.18632/oncotarget.7795)
Supplement: Supplementary file 1 [file oncotarget-07-21631-s001.pdf]

High LEF1 expression predicts adverse prognosis in chronic lymphocytic leukemia and may be targeted by ethacrynic acid

Supplementary Material

Supplement Table 1: Notch1 primers

Exon	Primer
HD-N/exon 26	Forward-5'-GGA AGG CGG CCT GAG CGT GTC-3'
	Reverse-5'-ATT GAC CGT GGG CGC CGG GTC-3'
HD-C/exon 27	Forward-5'-GCC TCA GTG TCC TGC GGC-3'
	Reverse-5'-GCA CAA ACA GCC AGC GTG TC-3'
TAD/exon 34	Forward-5'-GCT GGC CTT TGA GAC TGG C-3'
	Reverse-5'-GCT GAG CTC ACG CCA AGG T-3'
PEST/exon 34	Forward-5'-CAG ATG CAG CAG CAG AAC CTG-3'
	Reverse-5'-AAA GGA AGC CGG GGT CTC GT-3'

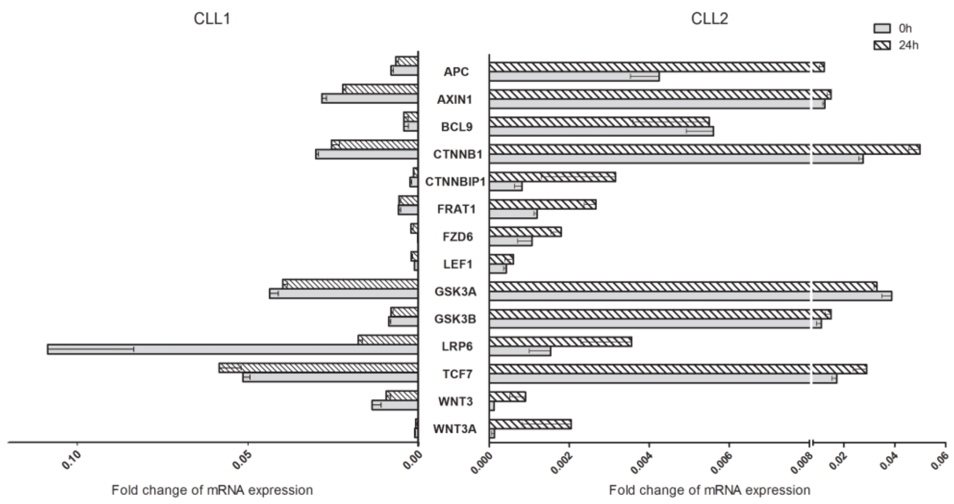

Supplement Figure 1: The effect of EA on the expressions of Wnt component genes in CLL cells. Primary cells from two CLL patients were collected and treated with 10μM EA for 24 hours. Realtime-PCR was employed to detect the mRNA expressions of Wnt component genes. The relative fold change was normalized against GAPDH.
